# Supplementary material for: Dietary patterns and associated factors among pregnant women in Ibadan, Nigeria: Evidence from Ibadan pregnancy cohort study
Source: PLoS One. 2022 Sep 15;17(9):e0273796. doi: 10.1371/journal.pone.0273796 (PMC9477303; doi:10.1371/journal.pone.0273796)
Supplement: Attachment — Submitted filename: RESPONSE TO THE REVIEWERS_plos_dietary.docx [file pone.0273796.s001.docx]

POINT BY POINT RESPONSE TO THE REVIEWERS COMMENTS

5. Review Comments to the Author

Reviewer #1: As you have described, prenatal nutrition impacts birth outcomes and is also an important factor that can potentially be modified. Your research will be beneficial in informing public health education, interventions, and policy.

- Thank you Sir

Overall, the paper is well written and succinctly describes your study methods and results.

- We thank the reviewer for the kind comment.

However minor editing of the paper is needed, as in some places words are missing or there are minor grammatical errors (including in lines 91, 103, 127 (unknown what the * references), 130, 158, 163, 211, 360, 372, 374, 40-408, 414, 416, 420, 427, 450).

- We are grateful to the reviewer for succinctly pointing our attention to these grammatical errors. The entire manuscript has been revised to ensure clarity in expression and avoid grammatical errors

Specific recommendations:
• Lines 167-168: Please provide another sentence or two describing how the dietary information was transformed and harmonized

We have included the following statements in lines 207 - 237 of the revised manuscript to described how dietary information was harmonized in the revised manuscript. Please the statement below

*“Details of the food and drink items in the FFQ and how they are classified into food groups are presented in Table 1. For each food or drink, participants reported the frequency of food consumption as follows: once daily, more than once daily (i.e. 2 -3 times daily): once weekly, more than once weekly (i.e. 2 -3 times weekly),): once monthly, more than once monthly (i.e. 2 -3 times monthly). The consumption frequency was harmonised into daily, weekly, monthly and rarely and transformed into the frequency of daily consumption.* ”

• Lines 218-224: To provide clarity for the reader, these results need to have consistency in the format of reporting, particularly related to use of the ( ) and [ ] and mentioning daily and weekly, for example use a format like this in reporting all the results in this paragraph:
o [294 (45.5% daily, 898 (51.4% weekly]

- Edited

• Page 18, table 5: Please define “Exotic Diet with alcohol” the term “Exotic Diet” is not described in the manuscript

- The correct description here is “Typical diet with alcohol”.
- We have changed “Exotic Diet with alcohol” to “Typical diet with alcohol” in Table 5 of the revised manuscrip.

• Lines 389-390: The study did not document that lower education and lower income caused higher alcohol consumption due to low health literacy regarding the adverse effects of alcohol, please redraft the sentence

- We have revised the sentence in lines 571 - 582 of the revised manuscript rephrases the discussion between alcohol intake and literacy.
- *Additionally, socioeconomic status had a negative association with this dietary pattern, i.e., women with low education and income had a higher consumption of this alcohol-based diet perhaps due to a lack of awareness of the adverse effects of alcohol consumption during pregnancy. A study in Uganda also reported that the availability of cheap alcoholic drinks and its free distribution during celebrations make alcoholic intake common among low income earning women (40)*

• Line 441: another potential bias is response bias, as the respondents may not have been entirely truthful or may have only wanted to report “good” eating habits in responding to the interviewers’ questions

- We have included the following sentence in line 704 -706 the revised manuscript to itemize this unique bias suggested by the reviewer. Thank you.

• Line 459-462: identify specific research/manuscript contributions of each individual author

- We are grateful to the reviewer for this suggestion. We have included the following statements in lines 710 -713 of the revised manuscript to itemize the contribution of each individual author. Please see the statement below;
- **Author’s contributions**
- *IAA designed and conducted the study and analyzed the data. IAA and APO interpreted the data and wrote the initial draft of the manuscript. IAA and APO reviewed and critically revised the manuscript. All authors read and approved the final manuscript.*
