# Supplementary material for: Dietary patterns and associated factors among pregnant women in Ibadan, Nigeria: Evidence from Ibadan pregnancy cohort study
Source: PLoS One. 2022 Sep 15;17(9):e0273796. doi: 10.1371/journal.pone.0273796 (PMC9477303; doi:10.1371/journal.pone.0273796)
Supplement: Attachment — Submitted filename: POINT BY POINT RESPONSE TO THE REVIEWERS_R2 dietary.docx [file pone.0273796.s002.docx]

POINT BY POINT RESPONSE TO THE REVIEWERS COMMENTS

6. Review Comments to the Author

Please use the space provided to explain your answers to the questions above. You may also include additional comments for the author, including concerns about dual publication, research ethics, or publication ethics. (Please upload your review as an attachment if it exceeds 20,000 characters)

Reviewer #1: Thank you for the revised manuscript and addressing the suggested comments. A number of edits were made, however because of the revisions the manuscript will again benefit from minor English editing.

I didn't complete a detailed language review of the entire manuscript, but here are some initial edits I identified in the revised manuscript and there may be others:

Line #93, believe the correct word is "other" vs. "over"

- Replaced with other *(line 92 page 4)*

Line #103, the verb should be "were" vs. "was"

- Replaced with were *(line 101 page 4)*

Line #106, delete "that *(line 104 page 4)*

- deleted
  Lines #138-139 and #141-143 seem to repeat the same information
- Lines #138-139 deleted *(line 134 page 5)*
